# Electrostatic repulsion between the cations of (1-methyl-1*H*-imidazole-κ*N* ^3^)(2,2′:6′,2′′-terpyridine-κ^3^ *N*,*N*′,*N*′′)platinum(II) perchlorate nitro­methane monosolvate prevents Pt⋯Pt inter­actions

**DOI:** 10.1107/S1600536811025475

**Published:** 2011-07-09

**Authors:** Matthew Akerman, Kate Akerman, Deogratius Jaganyi, Desigan Reddy

**Affiliations:** a School of Chemistry, University of KwaZulu-Natal, Private Bag X01, Pietermaritzburg 3209, South Africa

## Abstract

The reaction between [Pt(terpy)Cl]·2H_2_O (terpy = 2′,2′′:6′,2′′-terpyridine) and 1-methyl­imidazole (MIm) in the presence of two equivalents of AgClO_4_ in nitro­methane yields the title compound, [Pt(C_15_H_11_N_3_)(C_4_H_6_N_2_)](ClO_4_)_2_·CH_3_NO_2_. The dicationic complexes are arranged in a staggered configuration. The torsion angle subtended by the 1-methyl­imidazole ring relative to the terpyridine ring is 114.9 (5)°. Inter­molecular C—H⋯O inter­actions between the perchlorate anions and the H atoms of the terpy ligand are observed. Consideration of related phenyl­bipyridyl complexes of platinum(II), which are monocationic, leads to the conclusion that the electrostatic repulsion between the dicationic chelates prevents the formation of Pt⋯Pt inter­actions. These inter­actions are a common feature associated with the monocationic species.

## Related literature

For synthesis of the parent complex, chloro­(2,2′:6′,2"-ter­pyridine)­platinum(II)chloride dihydrate, [Pt(terpy)Cl]Cl·2H_2_O, see: Pitteri *et al.* (1995 ▶). For the structure of the aceto­nitrile solvate of the title Pt(II) chelate, see: Roszak *et al.* (1996 ▶) and for a structure, which was devoid of solvent in the lattice, see: Müller *et al.* (2007 ▶). For studies of the luminescent properties and inter­molecular Pt⋯Pt inter­actions of related compounds, see: Field *et al.* (2007 ▶). For a comprehensive review of Pt(II) terpyridines in general, see: Newkome *et al.* (2008 ▶). For Pt⋯Pt and Pt⋯π inter­actions in monocationic platinum(II) terpyridine and platinum(II) bipy complexes and the role they play in their unusual solid state emission properties, see: Connick *et al.* (1997 ▶); Field *et al.* (2003 ▶); Jaganyi & Reddy (2008 ▶).
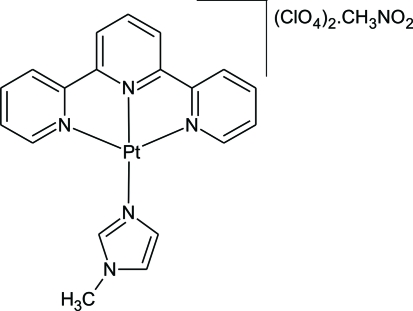


## Experimental

### 

#### Crystal data

[Pt(C_15_H_11_N_3_)(C_4_H_6_N_2_)](ClO_4_)_2_·CH_3_NO_2_
*M* *_r_* = 770.41
Monoclinic, 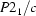

*a* = 16.389 (4) Å
*b* = 11.582 (5) Å
*c* = 14.538 (5) Å
β = 110.147 (5)°
*V* = 2590.7 (16) Å^3^
*Z* = 4
Mo *K*α radiation
μ = 5.69 mm^−1^
*T* = 296 K
0.60 × 0.60 × 0.60 mm

#### Data collection

Oxford Diffraction Xcalibur2 CCD diffractometer
Absorption correction: multi-scan (Blessing, 1995 ▶) *T*_min_ = 0.132, *T*_max_ = 0.132
17563 measured reflections
5102 independent reflections
3881 reflections with *I* > 2σ(*I*)
*R*_int_ = 0.053

#### Refinement

*R*[*F* ^2^ > 2σ(*F* ^2^)] = 0.045
*wR*(*F* ^2^) = 0.118
*S* = 0.98
5102 reflections
354 parameters
H-atom parameters constrained
Δρ_max_ = 1.59 e Å^−3^
Δρ_min_ = −2.64 e Å^−3^

### 

Data collection: *CrysAlis CCD* (Oxford Diffraction, 2008 ▶); cell refinement: *CrysAlis CCD*; data reduction: *CrysAlis RED* (Oxford Diffraction, 2008 ▶); program(s) used to solve structure: *SHELXS97* (Sheldrick, 2008 ▶); program(s) used to refine structure: *SHELXL97* (Sheldrick, 2008 ▶); molecular graphics: *WinGX* (Farrugia, 1999 ▶); software used to prepare material for publication: *WinGX* (Farrugia, 1999 ▶).

## Supplementary Material

Crystal structure: contains datablock(s) I, global. DOI: 10.1107/S1600536811025475/fi2109sup1.cif

Structure factors: contains datablock(s) I. DOI: 10.1107/S1600536811025475/fi2109Isup2.hkl

Additional supplementary materials:  crystallographic information; 3D view; checkCIF report

## Figures and Tables

**Table 1:** Hydrogen-bond geometry (Å, °)

*D*—H⋯*A*	*D*—H	H⋯*A*	*D*⋯*A*	*D*—H⋯*A*
C2—H2⋯O7^i^	0.93	2.51	3.21 (2)	132 (1)
C8—H8⋯O7^ii^	0.93	2.56	3.42 (2)	153 (1)
C9—H9⋯O6^iii^	0.93	2.54	3.34 (1)	144 (1)

Symmetry codes: (i) 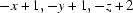; (ii) 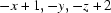; (iii) 
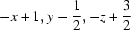
.

**Fig. 1.**
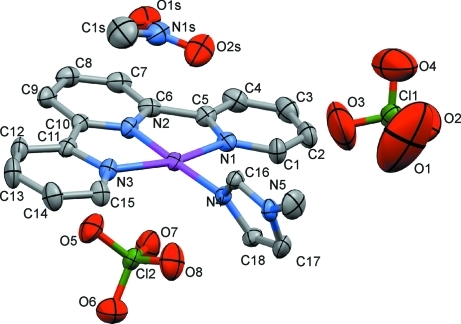
Molecular structure (50% probability surfaces) of [Pt(terpy)MIm](ClO4)2(CH3NO2). Hydrogen atoms have been omitted for clarity.

## supplementary crystallographic information

### Comment

Platinum(II) polypyridine complexes are an important class of compounds in many areas of inorganic chemistry (Newkome *et al.*, 2008). The most widely studied polypyridine ligands include 2,2'-bipyridine (bipy), 1,10-phenanthroline, 2,2':6',*N*''-terpyridine (terpy), 6-phenyl-2,2'-bipyridine and 2,2':6',2'':6'',2'''-quaterpyridine (Newkome *et al.*, 2008). The substitution kinetics of platinum(II) polypyridines have been extensively studied. This is largely due to their redox stability and relatively fast reactivity (Jaganyi and Reddy, 2008). To develop the understanding of the binding mode of the incoming 1-methylimidazole ligand in substitution kinetics, the single-crystal X-ray structure of the title compound was elucidated. Upon examination it was evident that the structure was devoid of any Pt···Pt or Pt···π interactions. These interactions are ubiquitous with monocationic platinum(II) terpyridine (Field *et al.*, 2007) and platinum(II) bipy complexes (Connick *et al.*, 1997) and are, in part, responsible for their unusual solid state emission properties (Field *et al.*, 2003).

Two variants of the title cation have been reported. Firstly, with an acetonitrile molecule in the lattice (Roszak *et al.*, 1996) and secondly, a structure, which was devoid of solvent in the lattice (Müller *et al.*, 2007). The title compound has a nitromethane molecule in the asymmetric unit.

The platinum(II) centre is nominally square planar with the tridentate terpy ligand and the monodentate 1-methylimidazole ligand, as expected for *d*^8^ platinum(II) complexes. The Pt— N1 and Pt— N3 bond distances are approximately equal, averaging 2.023 (8) Å. The Pt— N2 bond is 1.925 (5) Å. The Pt— N4 bond length is 2.013 (6) Å, these bond distances are equivalent to those previously reported. The MIm ligand is canted relative to the Pt(terpy) unit. The torsion angle defined by C(16)—N(4)—Pt—N(1) is 114.9 (5)°.This angle is equivalent to that reported by Roszak *et al.*, 1996. The same torsion angle reported by Müller *et al.*, 2007, is 107.2 (7)°. These data suggest that the identity of the solvent in the lattice is inconsequential in terms of its effect on the torsion angle of the ancillary ligand, but show that the presence of solvent influences the geometry of the cation. The platinum(II) chelates are arranged in a staggered configuration and are not parallel. Thus there are no Pt···Pt or Pt···π interactions in the solid state, which require interacting molecules to be parallel.

The crystal packing shows there to be no classical hydrogen bonding, however, there are unconventional hydrogen bonds between the perchlorate anions and the hydrogen atoms of the terpy ligand. The hydrogen bond parameters are given in Table 1.

### Experimental

The complex, chloro(2,2':6',2"-terpyridine)platinum(II)chloride dihydrate, [Pt(terpy)C1]C1.2H_2_O was synthesized using previously reported methods (Pitteri *et al.*, 1995). [Pt(terpy)Cl]Cl.2H_2_O (70 mg, 0.13 mmol) was dissolved in nitromethane (4 ml) and AgClO_4_ (54 mg, 0.26 mmol) was added to the stirred platinum solution at *ca*. 50 *^o^*C. The reaction mixture was stirred for 1 h over which time it turned a pale yellow in colour with a white silver(I)chloride precipitate. The precipitate was removed by filtration. One equivalent of 1-methylimidazole (11 mg, 0.13 mmol) was added to the filtrate, which immediately turned orange in colour. Orange crystals of [Pt(terpy)MIm](ClO_4_)_2_(CH_3_NO_2_) were grown *via* vapour diffusion of diethylether into the nitromethane solution of the desired product (yield 73%).

### Refinement

H atoms were refined riding with C—H (aromatic) distances of 0.93 Å and included in the refinement with Uiso = 1.2 Ueq.

### Crystal data

**Table:**

[Pt(C_15_H_11_N_3_)(C_4_H_6_N_2_)](ClO_4_)_2_·CH_3_NO_2_	*F*(000) = 1496
*M**_r_* = 770.41	*D*_x_ = 1.975 Mg m^−^^3^
Monoclinic, *P*2_1_/*c*	Mo *K*α radiation, λ = 0.71073 Å
Hall symbol: -P 2ybc	Cell parameters from 5102 reflections
*a* = 16.389 (4) Å	θ = 2.8–26.1°
*b* = 11.582 (5) Å	µ = 5.69 mm^−^^1^
*c* = 14.538 (5) Å	*T* = 296 K
β = 110.147 (5)°	Cubic, orange
*V* = 2590.7 (16) Å^3^	0.60 × 0.60 × 0.60 mm
*Z* = 4	

### Data collection

**Table:**

Oxford Diffraction Xcalibur2 CCD diffractometer	5102 independent reflections
Radiation source: fine-focus sealed tube	3881 reflections with *I* > 2σ(*I*)
graphite	*R*_int_ = 0.053
ω scans at fixed θ angles	θ_max_ = 26.1°, θ_min_ = 2.8°
Absorption correction: multi-scan (Blessing, 1995)	*h* = −20→20
*T*_min_ = 0.132, *T*_max_ = 0.132	*k* = −14→14
17563 measured reflections	*l* = −17→14

### Refinement

**Table:**

Refinement on *F*^2^	Primary atom site location: structure-invariant direct methods
Least-squares matrix: full	Secondary atom site location: difference Fourier map
*R*[*F*^2^ > 2σ(*F*^2^)] = 0.045	Hydrogen site location: inferred from neighbouring sites
*wR*(*F*^2^) = 0.118	H-atom parameters constrained
*S* = 0.98	*w* = 1/[σ^2^(*F*_o_^2^) + (0.082*P*)^2^] where *P* = (*F*_o_^2^ + 2*F*_c_^2^)/3
5102 reflections	(Δ/σ)_max_ = 0.001
354 parameters	Δρ_max_ = 1.59 e Å^−^^3^
0 restraints	Δρ_min_ = −2.64 e Å^−^^3^

### Special details

**Table:**

Geometry. All s.u.'s (except the s.u. in the dihedral angle between two l.s. planes) are estimated using the full covariance matrix. The cell s.u.'s are taken into account individually in the estimation of s.u.'s in distances, angles and torsion angles; correlations between s.u.'s in cell parameters are only used when they are defined by crystal symmetry. An approximate (isotropic) treatment of cell s.u.'s is used for estimating s.u.'s involving l.s. planes.
Refinement. Refinement of *F*^2^ against ALL reflections. The weighted *R*-factor *wR* and goodness of fit *S* are based on *F*^2^, conventional *R*-factors *R* are based on *F*, with *F* set to zero for negative *F*^2^. The threshold expression of *F*^2^ > 2σ(*F*^2^) is used only for calculating *R*-factors(gt) *etc*. and is not relevant to the choice of reflections for refinement. *R*-factors based on *F*^2^ are statistically about twice as large as those based on *F*, and *R*- factors based on ALL data will be even larger.

### Fractional atomic coordinates and isotropic or equivalent isotropic displacement parameters (Å^2^)

**Table:**

	*x*	*y*	*z*	*U*_iso_*/*U*_eq_	
C1	0.6697 (5)	0.4867 (7)	1.0042 (6)	0.059 (2)	
H1	0.6976	0.5318	0.9712	0.071*	
C1S	0.9475 (7)	0.0921 (12)	1.0904 (9)	0.118 (4)	
H1S1	0.9664	0.0286	1.1351	0.176*	
H1S2	0.9972	0.1356	1.0896	0.176*	
H1S3	0.9167	0.0633	1.0258	0.176*	
C2	0.6293 (6)	0.5379 (8)	1.0631 (6)	0.069 (2)	
H2	0.6308	0.6179	1.0693	0.082*	
C3	0.5873 (5)	0.4746 (9)	1.1124 (6)	0.071 (3)	
H3	0.5594	0.5097	1.1509	0.086*	
C4	0.5882 (5)	0.3568 (9)	1.1025 (6)	0.063 (2)	
H4	0.5614	0.3110	1.1363	0.076*	
C5	0.6272 (4)	0.3058 (7)	1.0446 (5)	0.0450 (17)	
C6	0.6301 (4)	0.1792 (8)	1.0298 (5)	0.0483 (18)	
C7	0.5995 (5)	0.0928 (9)	1.0705 (6)	0.060 (2)	
H7	0.5695	0.1096	1.1128	0.072*	
C8	0.6121 (5)	−0.0197 (8)	1.0502 (6)	0.061 (2)	
H8	0.5911	−0.0782	1.0798	0.073*	
C9	0.6548 (5)	−0.0487 (7)	0.9871 (5)	0.059 (2)	
H9	0.6620	−0.1253	0.9721	0.070*	
C10	0.6871 (4)	0.0424 (6)	0.9465 (5)	0.0435 (16)	
C11	0.7363 (4)	0.0341 (6)	0.8776 (5)	0.0452 (17)	
C12	0.7570 (5)	−0.0665 (7)	0.8412 (6)	0.062 (2)	
H12	0.7398	−0.1371	0.8590	0.074*	
C13	0.8024 (6)	−0.0627 (7)	0.7794 (7)	0.069 (2)	
H13	0.8190	−0.1310	0.7572	0.083*	
C14	0.8241 (5)	0.0410 (8)	0.7494 (6)	0.067 (2)	
H14	0.8527	0.0440	0.7042	0.080*	
C15	0.8030 (4)	0.1402 (7)	0.7870 (6)	0.0525 (19)	
H15	0.8195	0.2107	0.7684	0.063*	
C16	0.8566 (4)	0.4276 (6)	0.8775 (5)	0.0430 (16)	
H16	0.9022	0.3812	0.9149	0.052*	
C17	0.7844 (6)	0.5684 (7)	0.7859 (6)	0.061 (2)	
H17	0.7717	0.6360	0.7491	0.073*	
C18	0.7266 (5)	0.4945 (6)	0.8026 (5)	0.0475 (17)	
H18	0.6665	0.5028	0.7788	0.057*	
C19	0.9464 (5)	0.5765 (8)	0.8392 (7)	0.080 (3)	
H19A	0.9931	0.5231	0.8681	0.121*	
H19B	0.9556	0.6450	0.8786	0.121*	
H19C	0.9446	0.5962	0.7744	0.121*	
N1	0.6682 (4)	0.3704 (6)	0.9951 (4)	0.0453 (14)	
N1S	0.8913 (5)	0.1653 (8)	1.1213 (6)	0.072 (2)	
N2	0.6734 (4)	0.1509 (5)	0.9674 (4)	0.0429 (14)	
N3	0.7591 (4)	0.1392 (5)	0.8502 (4)	0.0438 (14)	
N4	0.7711 (4)	0.4065 (5)	0.8597 (4)	0.0399 (13)	
N5	0.8647 (4)	0.5235 (5)	0.8340 (4)	0.0469 (14)	
O1	0.941 (2)	0.7727 (17)	1.039 (3)	0.38 (2)	
O1S	0.8411 (5)	0.1229 (7)	1.1517 (5)	0.102 (2)	
O2	0.8371 (10)	0.7867 (13)	1.1015 (11)	0.203 (7)	
O2S	0.8951 (5)	0.2668 (7)	1.1098 (8)	0.114 (3)	
O3	0.8708 (12)	0.6230 (12)	1.0429 (10)	0.252 (8)	
O4	0.9562 (13)	0.7038 (14)	1.1756 (10)	0.261 (10)	
O5	0.4957 (4)	0.1229 (6)	0.7854 (5)	0.092 (2)	
O6	0.4009 (6)	0.2158 (7)	0.6530 (6)	0.104 (3)	
O7	0.3906 (7)	0.2504 (6)	0.8042 (7)	0.096 (3)	
O8	0.5069 (5)	0.3205 (8)	0.7608 (7)	0.109 (3)	
Cl1	0.89895 (18)	0.7258 (2)	1.08438 (18)	0.0666 (6)	
Cl2	0.45100 (13)	0.22848 (15)	0.75630 (14)	0.0472 (4)	
Pt	0.720986 (15)	0.27470 (2)	0.913208 (17)	0.03619 (12)	

### Atomic displacement parameters (Å^2^)

**Table:**

	*U*^11^	*U*^22^	*U*^33^	*U*^12^	*U*^13^	*U*^23^
C1	0.069 (5)	0.040 (5)	0.072 (5)	−0.007 (4)	0.030 (4)	−0.012 (4)
C1S	0.088 (8)	0.126 (12)	0.134 (10)	0.026 (8)	0.032 (8)	−0.005 (9)
C2	0.073 (6)	0.056 (6)	0.076 (6)	0.005 (5)	0.025 (5)	−0.022 (5)
C3	0.064 (6)	0.082 (7)	0.072 (5)	0.003 (5)	0.028 (5)	−0.020 (5)
C4	0.049 (5)	0.087 (8)	0.059 (5)	−0.011 (4)	0.025 (4)	−0.006 (4)
C5	0.030 (4)	0.052 (5)	0.054 (4)	−0.003 (3)	0.016 (3)	0.003 (3)
C6	0.032 (4)	0.066 (5)	0.045 (4)	−0.009 (4)	0.011 (3)	0.006 (4)
C7	0.043 (4)	0.076 (7)	0.055 (4)	−0.016 (4)	0.012 (4)	0.009 (4)
C8	0.050 (5)	0.070 (6)	0.057 (5)	−0.019 (4)	0.010 (4)	0.014 (4)
C9	0.059 (5)	0.044 (5)	0.061 (5)	−0.020 (4)	0.005 (4)	0.004 (4)
C10	0.034 (4)	0.040 (4)	0.047 (4)	−0.010 (3)	0.002 (3)	0.002 (3)
C11	0.035 (4)	0.041 (4)	0.054 (4)	0.000 (3)	0.008 (3)	0.004 (3)
C12	0.065 (5)	0.035 (4)	0.079 (6)	−0.003 (4)	0.018 (5)	−0.009 (4)
C13	0.066 (6)	0.040 (5)	0.107 (7)	−0.005 (4)	0.037 (5)	−0.023 (5)
C14	0.060 (5)	0.063 (6)	0.088 (6)	−0.006 (4)	0.038 (5)	−0.021 (5)
C15	0.036 (4)	0.051 (5)	0.074 (5)	−0.010 (3)	0.024 (4)	−0.011 (4)
C16	0.044 (4)	0.035 (4)	0.048 (4)	−0.003 (3)	0.012 (3)	0.004 (3)
C17	0.081 (6)	0.035 (4)	0.067 (5)	0.001 (4)	0.027 (5)	0.005 (4)
C18	0.044 (4)	0.044 (4)	0.051 (4)	0.009 (3)	0.012 (3)	0.005 (3)
C19	0.071 (6)	0.068 (6)	0.107 (7)	−0.032 (5)	0.038 (5)	0.006 (5)
N1	0.035 (3)	0.050 (4)	0.050 (3)	0.006 (3)	0.013 (3)	−0.004 (3)
N1S	0.051 (5)	0.068 (6)	0.082 (5)	−0.017 (4)	0.005 (4)	0.012 (4)
N2	0.032 (3)	0.039 (4)	0.057 (3)	−0.011 (2)	0.013 (3)	0.002 (3)
N3	0.031 (3)	0.036 (3)	0.065 (4)	−0.001 (2)	0.017 (3)	0.001 (3)
N4	0.043 (3)	0.030 (3)	0.046 (3)	0.001 (3)	0.014 (3)	−0.001 (2)
N5	0.048 (4)	0.037 (3)	0.058 (3)	−0.014 (3)	0.021 (3)	−0.006 (3)
O1	0.44 (4)	0.27 (2)	0.64 (5)	0.02 (2)	0.43 (4)	0.16 (2)
O1S	0.083 (5)	0.123 (7)	0.107 (5)	−0.020 (5)	0.041 (4)	0.026 (5)
O2	0.140 (11)	0.250 (16)	0.216 (15)	0.098 (11)	0.057 (10)	−0.020 (11)
O2S	0.060 (5)	0.095 (7)	0.164 (9)	−0.006 (4)	0.011 (5)	0.015 (6)
O3	0.39 (2)	0.153 (12)	0.218 (13)	−0.087 (13)	0.112 (14)	−0.097 (11)
O4	0.33 (2)	0.270 (18)	0.130 (10)	0.168 (17)	0.016 (12)	−0.018 (11)
O5	0.082 (5)	0.066 (4)	0.123 (6)	0.030 (4)	0.030 (4)	0.016 (4)
O6	0.098 (6)	0.116 (7)	0.084 (5)	0.006 (4)	0.013 (4)	0.006 (4)
O7	0.125 (7)	0.074 (5)	0.125 (7)	0.016 (4)	0.088 (6)	0.001 (4)
O8	0.095 (6)	0.076 (5)	0.167 (7)	−0.038 (5)	0.057 (5)	−0.016 (5)
Cl1	0.0814 (17)	0.0584 (13)	0.0685 (13)	0.0075 (12)	0.0366 (12)	−0.0004 (10)
Cl2	0.0475 (11)	0.0368 (9)	0.0627 (11)	0.0040 (8)	0.0258 (9)	0.0044 (8)
Pt	0.02992 (17)	0.03339 (17)	0.04422 (17)	−0.00357 (11)	0.01146 (12)	0.00027 (11)

### Geometric parameters (Å, °)

**Table:**

C1—N1	1.353 (10)	C14—C15	1.368 (11)
C1—C2	1.382 (11)	C14—H14	0.9300
C1—H1	0.9300	C15—N3	1.349 (9)
C1S—N1S	1.434 (13)	C15—H15	0.9300
C1S—H1S1	0.9600	C16—N5	1.307 (8)
C1S—H1S2	0.9600	C16—N4	1.356 (8)
C1S—H1S3	0.9600	C16—H16	0.9300
C2—C3	1.366 (13)	C17—C18	1.359 (11)
C2—H2	0.9300	C17—N5	1.363 (10)
C3—C4	1.372 (13)	C17—H17	0.9300
C3—H3	0.9300	C18—N4	1.357 (9)
C4—C5	1.356 (10)	C18—H18	0.9300
C4—H4	0.9300	C19—N5	1.452 (8)
C5—N1	1.365 (9)	C19—H19A	0.9600
C5—C6	1.485 (13)	C19—H19B	0.9600
C6—C7	1.345 (11)	C19—H19C	0.9600
C6—N2	1.372 (9)	N1—Pt	2.025 (6)
C7—C8	1.367 (13)	N1S—O1S	1.168 (9)
C7—H7	0.9300	N1S—O2S	1.192 (11)
C8—C9	1.374 (11)	N2—Pt	1.925 (5)
C8—H8	0.9300	N3—Pt	2.021 (6)
C9—C10	1.400 (10)	N4—Pt	2.013 (6)
C9—H9	0.9300	O1—Cl1	1.234 (16)
C10—N2	1.330 (9)	O2—Cl1	1.327 (11)
C10—C11	1.489 (10)	O3—Cl1	1.343 (12)
C11—C12	1.369 (11)	O4—Cl1	1.359 (14)
C11—N3	1.372 (9)	O5—Cl2	1.413 (7)
C12—C13	1.351 (12)	O6—Cl2	1.450 (8)
C12—H12	0.9300	O7—Cl2	1.415 (8)
C13—C14	1.366 (12)	O8—Cl2	1.393 (7)
C13—H13	0.9300		
			
N1—C1—C2	119.5 (8)	N5—C16—N4	109.3 (6)
N1—C1—H1	120.2	N5—C16—H16	125.3
C2—C1—H1	120.2	N4—C16—H16	125.3
N1S—C1S—H1S1	109.5	C18—C17—N5	106.0 (7)
N1S—C1S—H1S2	109.5	C18—C17—H17	127.0
H1S1—C1S—H1S2	109.5	N5—C17—H17	127.0
N1S—C1S—H1S3	109.5	N4—C18—C17	108.8 (7)
H1S1—C1S—H1S3	109.5	N4—C18—H18	125.6
H1S2—C1S—H1S3	109.5	C17—C18—H18	125.6
C3—C2—C1	122.0 (9)	N5—C19—H19A	109.5
C3—C2—H2	119.0	N5—C19—H19B	109.5
C1—C2—H2	119.0	H19A—C19—H19B	109.5
C2—C3—C4	117.0 (8)	N5—C19—H19C	109.5
C2—C3—H3	121.5	H19A—C19—H19C	109.5
C4—C3—H3	121.5	H19B—C19—H19C	109.5
C5—C4—C3	121.5 (8)	C1—N1—C5	119.3 (6)
C5—C4—H4	119.3	C1—N1—Pt	127.3 (5)
C3—C4—H4	119.3	C5—N1—Pt	113.4 (5)
C4—C5—N1	120.8 (8)	O1S—N1S—O2S	123.1 (10)
C4—C5—C6	124.4 (7)	O1S—N1S—C1S	118.9 (10)
N1—C5—C6	114.8 (6)	O2S—N1S—C1S	117.9 (9)
C7—C6—N2	118.1 (8)	C10—N2—C6	122.9 (6)
C7—C6—C5	129.6 (7)	C10—N2—Pt	119.1 (5)
N2—C6—C5	112.3 (6)	C6—N2—Pt	117.9 (5)
C6—C7—C8	120.4 (8)	C15—N3—C11	117.9 (7)
C6—C7—H7	119.8	C15—N3—Pt	128.5 (5)
C8—C7—H7	119.8	C11—N3—Pt	113.6 (5)
C7—C8—C9	121.9 (8)	C16—N4—C18	106.5 (6)
C7—C8—H8	119.1	C16—N4—Pt	126.5 (4)
C9—C8—H8	119.1	C18—N4—Pt	127.0 (5)
C8—C9—C10	116.8 (8)	C16—N5—C17	109.4 (6)
C8—C9—H9	121.6	C16—N5—C19	125.4 (7)
C10—C9—H9	121.6	C17—N5—C19	125.1 (7)
N2—C10—C9	119.9 (7)	O1—Cl1—O2	118.1 (12)
N2—C10—C11	112.8 (6)	O1—Cl1—O3	108.6 (15)
C9—C10—C11	127.3 (7)	O2—Cl1—O3	112.9 (11)
C12—C11—N3	121.0 (7)	O1—Cl1—O4	106.3 (19)
C12—C11—C10	125.3 (7)	O2—Cl1—O4	103.3 (9)
N3—C11—C10	113.7 (6)	O3—Cl1—O4	106.7 (10)
C13—C12—C11	119.7 (8)	O8—Cl2—O5	112.5 (6)
C13—C12—H12	120.2	O8—Cl2—O7	113.6 (5)
C11—C12—H12	120.2	O5—Cl2—O7	112.7 (5)
C12—C13—C14	120.4 (8)	O8—Cl2—O6	104.8 (5)
C12—C13—H13	119.8	O5—Cl2—O6	105.8 (5)
C14—C13—H13	119.8	O7—Cl2—O6	106.6 (6)
C13—C14—C15	118.7 (8)	N2—Pt—N4	178.5 (2)
C13—C14—H14	120.6	N2—Pt—N3	80.7 (3)
C15—C14—H14	120.6	N4—Pt—N3	100.6 (2)
N3—C15—C14	122.3 (8)	N2—Pt—N1	81.5 (3)
N3—C15—H15	118.9	N4—Pt—N1	97.2 (2)
C14—C15—H15	118.9	N3—Pt—N1	162.2 (2)
			
N1—C1—C2—C3	−0.3 (14)	C7—C6—N2—Pt	−177.7 (5)
C1—C2—C3—C4	1.2 (14)	C5—C6—N2—Pt	0.3 (7)
C2—C3—C4—C5	−1.5 (13)	C14—C15—N3—C11	0.8 (11)
C3—C4—C5—N1	1.0 (12)	C14—C15—N3—Pt	−179.5 (6)
C3—C4—C5—C6	−179.5 (7)	C12—C11—N3—C15	−0.5 (10)
C4—C5—C6—C7	−2.5 (12)	C10—C11—N3—C15	−179.3 (6)
N1—C5—C6—C7	177.0 (7)	C12—C11—N3—Pt	179.8 (6)
C4—C5—C6—N2	179.8 (7)	C10—C11—N3—Pt	1.0 (7)
N1—C5—C6—N2	−0.6 (8)	N5—C16—N4—C18	0.1 (7)
N2—C6—C7—C8	0.4 (11)	N5—C16—N4—Pt	−177.2 (4)
C5—C6—C7—C8	−177.1 (7)	C17—C18—N4—C16	0.0 (8)
C6—C7—C8—C9	−0.9 (12)	C17—C18—N4—Pt	177.2 (5)
C7—C8—C9—C10	1.7 (11)	N4—C16—N5—C17	−0.1 (8)
C8—C9—C10—N2	−1.9 (10)	N4—C16—N5—C19	177.6 (7)
C8—C9—C10—C11	179.2 (7)	C18—C17—N5—C16	0.0 (9)
N2—C10—C11—C12	−177.8 (7)	C18—C17—N5—C19	−177.7 (7)
C9—C10—C11—C12	1.1 (12)	C10—N2—Pt—N3	2.5 (5)
N2—C10—C11—N3	0.9 (9)	C6—N2—Pt—N3	179.6 (5)
C9—C10—C11—N3	179.8 (6)	C10—N2—Pt—N1	−177.0 (5)
N3—C11—C12—C13	1.7 (12)	C6—N2—Pt—N1	0.0 (5)
C10—C11—C12—C13	−179.7 (7)	C16—N4—Pt—N3	−64.4 (6)
C11—C12—C13—C14	−3.2 (14)	C18—N4—Pt—N3	119.0 (6)
C12—C13—C14—C15	3.4 (14)	C16—N4—Pt—N1	114.9 (5)
C13—C14—C15—N3	−2.2 (13)	C18—N4—Pt—N1	−61.7 (6)
N5—C17—C18—N4	0.0 (8)	C15—N3—Pt—N2	178.5 (7)
C2—C1—N1—C5	−0.3 (11)	C11—N3—Pt—N2	−1.8 (4)
C2—C1—N1—Pt	179.4 (6)	C15—N3—Pt—N4	−2.2 (7)
C4—C5—N1—C1	0.0 (11)	C11—N3—Pt—N4	177.4 (5)
C6—C5—N1—C1	−179.6 (6)	C15—N3—Pt—N1	−179.9 (7)
C4—C5—N1—Pt	−179.8 (6)	C11—N3—Pt—N1	−0.2 (10)
C6—C5—N1—Pt	0.6 (7)	C1—N1—Pt—N2	179.9 (6)
C9—C10—N2—C6	1.5 (10)	C5—N1—Pt—N2	−0.4 (5)
C11—C10—N2—C6	−179.5 (6)	C1—N1—Pt—N4	0.6 (7)
C9—C10—N2—Pt	178.4 (5)	C5—N1—Pt—N4	−179.7 (5)
C11—C10—N2—Pt	−2.5 (8)	C1—N1—Pt—N3	178.3 (7)
C7—C6—N2—C10	−0.7 (10)	C5—N1—Pt—N3	−2.0 (10)
C5—C6—N2—C10	177.2 (6)		

### Hydrogen-bond geometry (°)

**Table:**

*D*—H···*A*
—···
—···
—···
